# Diurnal influences on electrophysiological oscillations and coupling in the dorsal striatum and cerebellar cortex of the anesthetized rat

**DOI:** 10.3389/fnsys.2014.00145

**Published:** 2014-09-15

**Authors:** Ariana Frederick, Jonathan Bourget-Murray, C. Andrew Chapman, Shimon Amir, Richard Courtemanche

**Affiliations:** ^1^Center for Studies in Behavioral Neurobiology/FRQS Groupe de Recherche en Neurobiologie Comportementale, Concordia UniversityMontreal, QC, Canada; ^2^Department of Biology, Concordia UniversityMontreal, QC, Canada; ^3^M.D., C.M. Program, Faculty of Medicine, McGill UniversityMontreal, QC, Canada; ^4^Department of Psychology, Concordia UniversityMontreal, QC, Canada; ^5^Department of Exercise Science, Concordia UniversityMontreal, QC, Canada

**Keywords:** local field potential oscillation, coherence, dopamine, circadian, urethane

## Abstract

Circadian rhythms modulate behavioral processes over a 24 h period through clock gene expression. What is largely unknown is how these molecular influences shape neural activity in different brain areas. The clock gene *Per2* is rhythmically expressed in the striatum and the cerebellum and its expression is linked with daily fluctuations in extracellular dopamine levels and D2 receptor activity. Electrophysiologically, dopamine depletion enhances striatal local field potential (LFP) oscillations. We investigated if LFP oscillations and synchrony were influenced by time of day, potentially via dopamine mechanisms. To assess the presence of a diurnal effect, oscillatory power and coherence were examined in the striatum and cerebellum of rats under urethane anesthesia at four different times of day zeitgeber time (ZT1, 7, 13 and 19—indicating number of hours after lights turned on in a 12:12 h light-dark cycle). We also investigated the diurnal response to systemic raclopride, a D2 receptor antagonist. Time of day affected the proportion of LFP oscillations within the 0–3 Hz band and the 3–8 Hz band. In both the striatum and the cerebellum, slow oscillations were strongest at ZT1 and weakest at ZT13. A 3–8 Hz oscillation was present when the slow oscillation was lowest, with peak 3–8 Hz activity occurring at ZT13. Raclopride enhanced the slow oscillations, and had the greatest effect at ZT13. Within the striatum and with the cerebellum, 0–3 Hz coherence was greatest at ZT1, when the slow oscillations were strongest. Coherence was also affected the most by raclopride at ZT13. Our results suggest that neural oscillations in the cerebellum and striatum, and the synchrony between these areas, are modulated by time of day, and that these changes are influenced by dopamine manipulation. This may provide insight into how circadian gene transcription patterns influence network electrophysiology. Future experiments will address how these network alterations are linked with behavior.

## Introduction

Local field potential (LFP) oscillations are recorded when rhythmic fluctuations in membrane potentials synchronize within a nearby group of cells. These oscillations can provide a mechanism for the storage and transfer of information by coordinating the neuronal activity between distant networks (Başar et al., 2001; Buehlmann and Deco, 2010; Uhlhaas et al., 2010). The many ion channels involved in maintaining the membrane potential and in generating post-synaptic potentials, along with neurotransmitter modulation, influence the dominant oscillatory parameters within neural networks (Hutcheon and Yarom, 2000; Buzsáki and Draguhn, 2004). In the suprachiasmatic nucleus (SCN), a strong influence on many of these cellular processes is the circadian expression of proteins that regulate neuron function and local neural network connections (Herzog, 2007; Colwell, 2011). Circadian protein expression shows distinct phase relationships throughout widespread areas of the brain, including the striatum and cerebellum (Namihira et al., 1999; Rath et al., 2012; Harbour et al., 2013), however, little is known about the role of circadian protein expression in regulating neuron function in these areas. In this paper, we examine how striatal and cerebellar networks may be under the influence of circadian rhythms. The role of the cerebellum in the context of circadian rhythms has scarcely been studied, however it has been shown to share a role with the dorsal striatum in regulating locomotor activity patterns under restricted feeding, a behavior that is modified by circadian genes (Mendoza et al., 2010; Verwey and Amir, 2012). Furthermore, there is an interest in the subcortical connectivity between these two areas and their cooperative contributions to motor control (Middleton and Strick, 2000; Bostan and Strick, 2010). In order to understand the neurophysiological changes associated with the circadian modulation influencing the striatum and cerebellum, we simultaneously recorded LFPs in these two areas and examined concurrent oscillations and their coherence.

Within the striatal networks, and the granule cell layer of the posterior lobe of the cerebellum of rats and primates, LFP oscillations around 5–30 Hz are present during a variety of tasks (Courtemanche et al., 2002, 2013; Berke et al., 2004; DeCoteau et al., 2007b). These oscillations, and their synchrony with more distant brain areas, are modulated under behavioral conditions such as sensorimotor associations, learning, reward expectancy and sleep (Destexhe et al., 1999; Steriade, 2003; Buzsáki, 2006; Thorn and Graybiel, 2014). Anesthetics, such as urethane, shape the LFPs of cortical and subcortical structures into predominant slow wave activity, inducing a sleep-like state in the LFP that shows widespread synchrony across many brain areas (Magill et al., 2004; Clement et al., 2008; Ros et al., 2009; Sharma et al., 2010). In this study, we used this oscillation-permissive state to evaluate the circadian expression of oscillatory network activity.

Both under anesthesia and in awake behaving animals, dopamine changes can induce important modifications to striatal oscillations (Walters et al., 2007; Lemaire et al., 2012). Dopamine depletion causes an increase in the power of oscillations below 30 Hz and an increase in coherence of these oscillations with the cerebral cortex and throughout the basal ganglia (Brown et al., 2001; Sharott et al., 2005). This is accompanied by a decrease in power of the oscillations above 70 Hz. Results from animal models that use dopamine antagonists or midbrain dopaminergic lesions are consistent with the findings in Parkinson’s disease in humans, where abnormal oscillations can be normalized with dopamine agonists or dopamine-related treatments such as L-DOPA (Brown et al., 2001; Rivlin-Etzion et al., 2006; Ballion et al., 2009), further demonstrating the specific role of dopamine in modulating striatal oscillations.

The dopaminergic system is under the influence of circadian rhythmicity. Endogenous extracellular dopamine levels show cyclic fluctuations throughout the day and peak during the middle of the dark phase in rodents (Owasoyo et al., 1979; Hood et al., 2010; Ferris et al., 2014). Furthermore, activation of D2 dopamine receptors has been linked with normal expression of *Per*2, one of the core circadian genes, in the rat dorsal striatum (Hood et al., 2010; Gravotta et al., 2011). *Per2* is involved in regulating daily physiological and behavioral cycles and is found in most tissues throughout the body (Albrecht et al., 2007). However, it is unclear how its rhythmic expression leads to such behavioral changes. Vesicle transporter activity throughout the brain (Darna et al., 2009) and changes in hippocampal synaptic plasticity (Chaudhury et al., 2005) exhibit diurnal variations; similarly, modulation of neurotransmitter function by the circadian clock provide potential ways through which circadian molecular mechanisms may be translated into diurnal electrophysiological and behavioral changes.

A limited number of studies have assessed the electrophysiological correlates of the circadian cycle downstream of the SCN, the master circadian clock, or its direct connections (Guilding and Piggins, 2007). Mordel et al. (2013) assessed diurnal variations of *in vitro* Purkinje cell firing and spontaneous inhibitory post-synaptic potentials, but they found that these measures were not modulated in a circadian manner. Restrictive feeding influences the rhythm of *Per*2 expression in the Purkinje and granule cell layers (Mendoza et al., 2010). There is limited evidence that dopamine levels are also rhythmic in the cerebellum (Owasoyo et al., 1979) but little more is known about circadian modulation of cerebellar physiology; including the functional role of clock genes or their regulatory mechanisms. Clock gene expression may help to organize the activity of striatal and cerebellar networks through mechanisms that affect the generation and synchronization of LFP oscillations. Such oscillations, recorded under urethane anesthesia, can provide information about the local activity and the neural synchronization between distant brain structures.

The purpose of this study was to determine if there are diurnal changes in LFP oscillations and coherence in the rat dorsal striatum and cerebellar cortex. Furthermore, since *Per*2 rhythms in the striatum are responsive to D2 receptor antagonists and not D1 antagonists (Hood et al., 2010), we also explored the effects of raclopride, a D2 antagonist, at different times of day. Given the endogenous rhythm of dopamine in the striatum, we hypothesized that circadian electrophysiological correlates would be expressed through diurnal changes. These changes could be seen in the potency of the LFP oscillations in each area, and in their synchrony. The basal ganglia and cerebellum show a cooperative role in sensorimotor control (Doya, 2000), and in their regulation of locomotor activity patterns related to circadian timing. These areas can interact through the cerebral cortex (Middleton and Strick, 2000) and via subcortical pathways (Ichinohe et al., 2000; Hoshi et al., 2005; Bostan et al., 2010; Bostan and Strick, 2010). In order to identify their network interactions, we thus measured the LFP coherence between distant electrodes (DeCoteau et al., 2007a; Thorn et al., 2010; Frederick et al., 2013). The variables thus sought were the diurnal effects in (1) the power of LFP oscillations; (2) the LFP coherence within and across the striatum and cerebellar cortex; and also (3) if raclopride would induce an alteration in the circadian pattern of oscillations or coherence.

## Materials and methods

### Animals

Sixteen Sprague-Dawley male rats (Charles River, St-Constant, QC) 300–400 g in size were used for the experiment. Rats were housed separately in cages equipped with running wheels and kept in individual lightproof and sound-attenuated cabinets. Prior to electrophysiological recordings, rats were maintained on a 12:12 h light-dark cycle for a minimum of 2 weeks. Rats were assigned randomly to groups designated by four different target experimental times, corresponding to zeitgeber time (ZT) 1, 7, 13 and 19, where ZT0 represents when lights were turned on and ZT12 when lights were turned off. Light cycles were adjusted for each animal so that the target experimental time occurred at 1:30 pm for all animals. Environmental conditions were maintained at a constant temperature and rats were provided *ad libitum* access to tap water and laboratory chow throughout the entire entrainment period. To confirm that rats had adjusted to their light cycles, wheel-running activity was monitored continuously using the VitalView software (Mini Mitter, Bend, OR). All experimental procedures followed the guidelines of the Canadian Council on Animal Care and were approved by the Concordia University Animal Research Ethics Committee.

### Drug treatment and recordings

Surgical preparation for each animal was scheduled such that the animal’s targeted circadian time occurred mid-way through the electrophysiological recording session. LFP recordings were obtained both before and after administration of raclopride. At the start of the recording procedures (ZT time – ~4 h) the rats were anesthetized with a 5% isoflurane and 95% oxygen mixture until placement of a catheter into the jugular vein. They were then transferred to urethane anesthesia (0.8 g/ml), introduced through the jugular catheter in 1.0 ml boluses as needed. Verification of depth of anesthesia was monitored throughout the experiment by lack of a foot-pinch reflex. In 11 animals, transcardial heartbeat signals were also recorded along with LFPs using subdermal electrodes inserted at the level of the axillar forelimb fossa. Rats were placed in a stereotaxic apparatus and body temperature was maintained at 37°C, monitored by a rectal thermometer. Throughout the experiment, the eyes of the animal were covered to prevent light exposure from influencing changes in the SCN.

Four pairs of tungsten microelectrodes (1.0–1.2 MΩ; FHC, Bowdoin, ME) were used, and the difference in depth within each pair of electrodes was set to 0.5 mm. Electrodes were attached to microdrives, held by 2 modified head stages one for the striatal electrodes and one for the cerebellar electrodes, which were anchored to the stereotaxic apparatus by custom built arms. Two pairs of striatal electrodes were used, one placed in the medial dorsal striatum [0.48 mm AP, 1.40 mm ML, 4.70 mm DV; (Paxinos and Watson, 1998)] and another placed in the lateral dorsal striatum (0.48 mm AP, 5.40 mm ML, 5.00 mm DV). Two pairs of electrodes were also inserted into the cerebellar cortex, separated by approximately 2 mm, in the contralateral Crus2 or Paramedian lobule of the cerebellar cortex. Access to Crus2/Paramedian lobule was gained through a craniotomy roughly 2.5 mm in diameter in the occipital bone, located 2–3 mm lateral from the midline. Crus2 was then identified visually and electrodes were inserted at a 45° angle to a depth of about 0.5 mm. The electrode position was adjusted until the granule cell layer was identified by characteristic multiunit activity and LFP oscillatory activity was identified on an oscilloscope. The 0.5 mm distance between electrodes within a pair suggests that both electrodes were not necessarily in the granule cell layer; this distance allowed for a comparison of nearby circuit activity with a strong granule cell layer influence, given the density of the granule cell layer and the electrode impedance. The nearby comparison could then be contrasted with the 2 mm distance of the second pair. All signals were grounded to stainless steel screws anchored to the skull on the contralateral side and referenced to a 16 gauge circular steel wire placed either in the cerebral cortex approximately 5 mm posterior to the striatal electrodes or on the unrecorded side of the cerebellum.

Electrophysiological signals were collected with a Neuralynx Cheetah system (Neuralynx, Bozeman, MT) at a sampling rate of 30,300 Hz and band-pass filtered between 0.1 and 9000 Hz. LFPs were then extracted from this signal with a 300-Hz low-pass filter. A typical recording session began with a first set of recordings, including 5 samples of 120 s separated by an interval of 90 s without recording, following which raclopride tartrate (Sigma-Aldrich, Oakville, ON) was administered via intraperitoneal injection (1 mg/kg dissolved in distilled water at 1 μg/ml). After a 15 min waiting period, 5 additional 120 s samples were recorded. Raclopride was administered at a dosage shown to induce akinesia and prevent operant conditioning, and to have effects on neural activity in the striatum as early as 5 min following intraperitoneal injection (Fowler and Liou, 1998; Dejean et al., 2011). Even though the animals were heated between recordings, a drop in body temperature sometimes occurred in the later recording periods; therefore, the second recording periods both before and after raclopride administration were used for analysis, since these showed stability across all subjects.

### Tissue staining

Following recordings, the brain was lesioned using a lesion maker (Grass Products, Warwick, RI) to mark electrode placement (300 μA for 30 s). Rats were then perfused with 300 ml cold 0.9% saline followed by 300 ml cold 4% paraformaldehyde in 0.1M phosphate buffer. Brains were extracted and kept overnight at 4°C in a 4% paraformaldehyde solution then dehydrated in phosphate buffered saline (PBS) with 30% sucrose. The cerebrum was sliced coronally and the cerebellum was sliced horizontally into 50 μm sections on a cryostat and mounted directly onto slides. Sections were stained using cresyl violet and lesions were located using light microscopy.

### Electrophysiological data processing

Raw microelectrode data was downsampled to 2020 Hz using Neuralynx software and off-line analysis of recorded data was conducted using custom Matlab (MathWorks, Natick, MA) functions and scripts. Once in Matlab, the data was low-pass filtered at 300 Hz and structured into 2-s windows. To analyze the rhythmicity content of the low-passed signal, Fast Fourier Transform (FFT) was calculated on each 2-s window. The following bands were used for analysis: (1) (0.1–3 Hz, delta); (2) (3–8 Hz, theta); (3) (8–13 Hz, alpha); (4) (13–30 Hz, beta); and (5) (30–55 Hz, gamma). The power spectrum was integrated within those bands, and then averaged for the 60 windows, providing one value per recording period. Coherence between LFP pairs was also calculated on each 2-s window and values within the same frequency bands were also averaged per recording period. The average FFT and coherence of each period was entered into the statistical analyses.

In order to establish the percentage of time where slow (0–3 Hz) vs. 3–8 Hz oscillations predominate, the integrated FFT power for each band was compared for each 2-s time window. Plotting those values in time for each recording period, we identified the power level around which the dominant power between 0–3 Hz and 3–8 Hz was alternating. To compute this, we calculated the difference between the 0–3 and 3–8 Hz integrated power and took the mean of the values where this difference equaled 0 ± 10 of the total percent FFT power. This difference maintained a narrow range while still including enough data points to normalize, thus providing a baseline for each recording session, from which a threshold could be calculated. This mean +20% value determined if a window had strong 0–3 Hz activity, while the mean –15% determined if a window had strong 3–8 Hz activity. These thresholds were chosen empirically as the most conservative levels where the 0–3 Hz or 3–8 Hz oscillations could be detected across multiple recording sessions. The number of windows that fell above the threshold was calculated for each band, and then divided by the total number of windows to get a value expressed as a percentage.

To avoid duplication of data, spindles were analyzed for one electrode in each of the MStr and LStr pairs of electrodes and for one electrode placed in the cerebellum. The average of the number of spindles detected from the two striatal electrodes was then used for statistical analysis. A detectable spindle was defined as an oscillation within 8–20 Hz that occurred at the peak of the slow wave. Spindles were identified by first band-pass filtering the LFP signal between 0–3 Hz to identify the slow oscillation phase. The LFP was also filtered between 8–20 Hz, and this filtered signal was rectified, and convolved with a 14-Hz (71 ms) window. These 8–20 Hz filtered and rectified product-values were *Z*-scored and a threshold was set at 2 standard deviations. Spindles were detected when the 8–20 Hz signal reached above this threshold value within ±0.25 s of the peak of a slow wave oscillation. An example is shown in Figure 5A.

**Figure 1:**
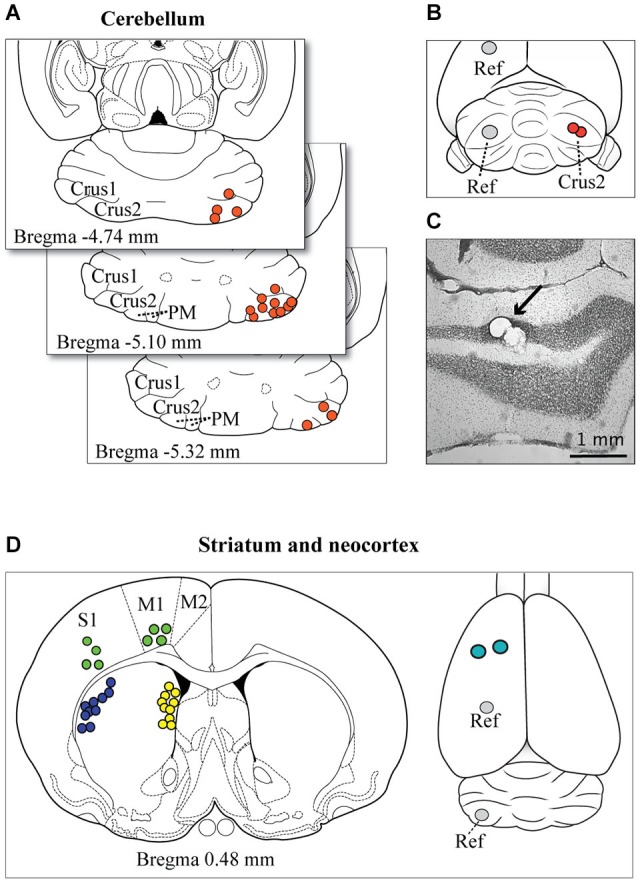
**Microelectrode tip localizations and reconstructions**. **(A)** The representative spread of identified electrode tip locations in the cerebellum are shown (filled circles) on horizontal sections taken from the atlas of Paxinos and Watson (1998). **(B)** Example of the target location of the two insertion points of the microelectrodes on the surface of the exposed cerebellar cortex (red circles). Cerebellar recordings were obtained on the right side of the brain, and the reference wire (Ref) was positioned in the opposite hemisphere in either the left cerebellar cortex or more rostrally in the parietal cortex. **(C)** A photomicrograph shows the location of microelectrode tip lesions in the granule cell layer of Crus2 (arrow). Most electrode tips were located in the granule cell layer or within close proximity. **(D)** The panel at left shows a representative spread of the location, based on atlas reconstructions, of identified microelectrode tips in the striatum (dorsolateral, blue circles; medial, yellow circles) and neocortex (green circles). The panel at right shows the optional locations of the reference wires (Ref) and the insertion points for cortical and striatal electrodes (teal circles).

**Figure 2:**
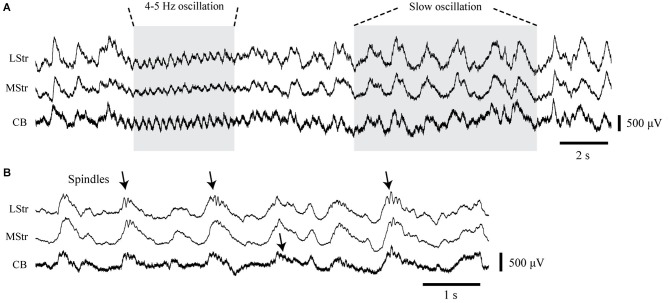
**LFP activity in the striatum and cerebellum under urethane anesthesia**. **(A)** Simultaneously recorded LFPs from the dorsolateral striatum (LStr), medial striatum (MStr), and cerebellar cortex (CB) containing concurrent periods of 4–5 Hz oscillations and slow 1 Hz oscillations. **(B)** Recordings at an expanded time scale obtained from another recording period show spindle-like activity in association with slow waves (arrows).

**Figure 3:**
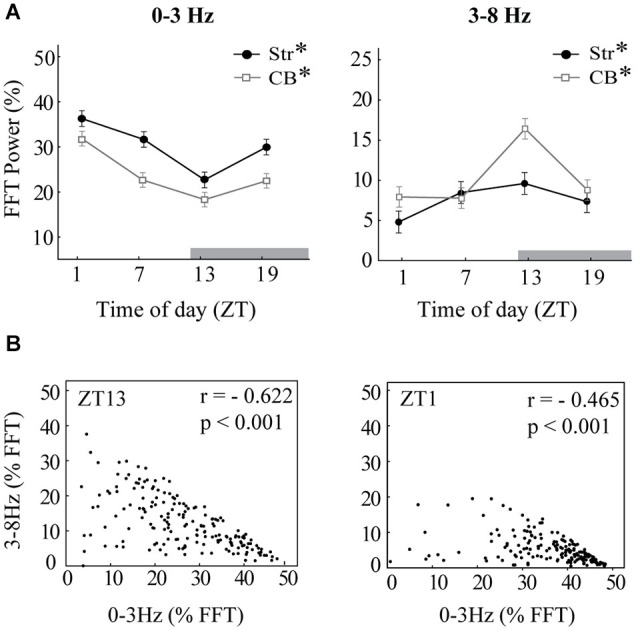
**Diurnal variation in oscillations. (A)** The mean integrated power of 0–3 Hz oscillations (left) and 3–8 Hz oscillations (right) is shown for each time of day at baseline, before raclopride administration, expressed as a percentage of the total power spectrum (0–55 Hz). Time of day (ZT) is expressed in hours after onset of light. Note that the dark period began at ZT12 (gray bar). Asterisks indicate significant effects of ZT (*p* < 0.05). **(B)** Scatterplots indicate strong negative correlations between the power of 0–3 Hz oscillations and 3–8 Hz oscillations. Each dot represents the integrated power within each of the 2-s windows that compose the baseline recordings. The panel at left shows an example from ZT13 that includes both 1 Hz oscillations and 4–5 Hz oscillations. The panel at right shows an example from ZT1 that contains mainly 1 Hz oscillations. Both examples are from striatal LFPs, though cerebellar LFPs showed a similar correlation.

**Figure 4:**
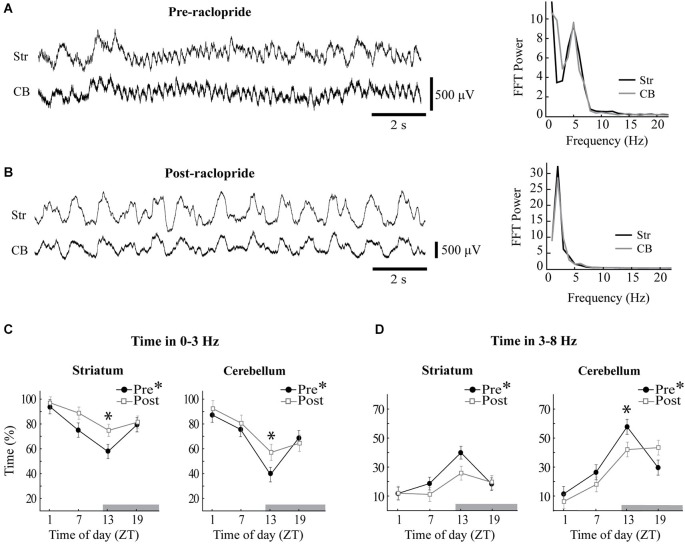
**Raclopride enhances slow oscillations and reduces 3–8 Hz oscillations at ZT13**. Sample LFPs obtained from the striatum (Str) and the cerebellum (CB) before **(A)** and after **(B)** systemic administration of raclopride. The baseline recording shows extended periods of 5 Hz oscillations in both sites, which were replaced by a strong 2 Hz oscillation following raclopride. Power spectra at right correspond to the LFPs displayed at left and reflect similar spectral content in striatal and cerebellar recordings.** (C,D)** The percentage of time that each recording site spent within 0–3 Hz **(C)** or 3–8 Hz **(D)** oscillations was calculated before (filled circles) and after (open squares) raclopride administration. Asterisks in the figure legend indicates a main effect of ZT at baseline (pre-raclopride), asterisks above the data points indicate significant effects of raclopride in the Tukey *post hoc* analysis, *p* < 0.05.

**Figure 5:**
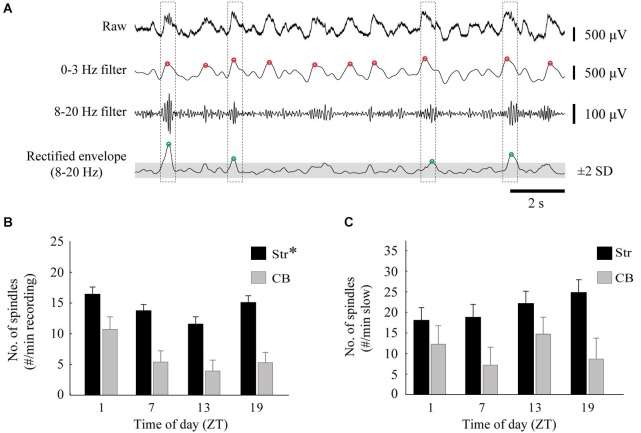
**Diurnal variation in spindles**. **(A)** An example of the spindle detection method on a striatal recording. Spindles were identified by low-pass filtering the raw signal at 0–3 Hz to locate the peaks of slow oscillations (red circles), and also band-pass filtered at 8–20 Hz. The 8–20 Hz signal was rectified and peaks 2 standard deviations above the mean (gray area) of the smoothed envelope were located (green circles). A spindle was identified when the peaks of the slow oscillations and 8–20 Hz activity occurred within 0.5 s of each other (dashed boxes). **(B)** The average number of spindles per minute, calculated from two striatal (one MStr, one LStr) and one cerebellar LFP, for each time of day (asterisk indicates a main effect of ZT, *p* < 0.05). **(C)** The average number of spindles, normalized to the percentage of time that the signal spent in slow-wave activity.

### Neocortical recordings

In order to verify if coherence between the striatum and cerebellar cortex under urethane anesthesia involves neocortical connections, four additional recording sessions were done with neocortical electrodes added, one at each time of day (ZT1, 7, 13 and 19). The same electrophysiological set-up was used as described above, but instead of placing two pairs of electrodes in the striatum, one electrode from each pair was stopped in the overlying neocortex, corresponding to the sensorimotor cortex. These recordings were pooled together with the rest of the dataset to provide an overall description of synchrony between the neocortex, the striatum and the cerebellum. Coherence within each frequency band was analyzed as above, however the trend was similar across all bands, so we report below the 0.1–8 Hz and overall coherence from 0.1 to 55 Hz.

### Statistical analysis

Statistical tests were performed using Statistica 10 (StatSoft, Tulsa, OK) or in Matlab. All results are presented as the mean ± the standard error of the mean (SEM). The second recording period before and after raclopride showed the most stability with body temperature; so all further analysis was performed on these two periods. The dependent variables of oscillatory power and coherence (for each frequency band) were statistically assessed for two different analyses. Circadian effect on baseline recordings was assessed by one-way repeated measures ANOVA, with time-of-day as the independent variable. The circadian effects of raclopride assessed the interaction between pre-post raclopride conditions and time-of day using two-way repeated-measures ANOVAs. Results were considered significant at *p* < 0.05. *Post-hoc* comparisons used Tukey tests. For identifying the relationship between 0–3 Hz and 3–8 Hz power across windows, linear correlation (*p* < 0.05) was used.

## Results

### *In vivo* recordings

LFP recordings were obtained from 16 urethane-anesthetized animals (4 animals × 4 ZT). There was no effect of either time-of-day or pre-post raclopride on the heart rate (ZT, *F*_(3,7)_ = 0.14, *p* = 0.93, pre-post, *F*_(1,7)_ = 0.14, *p* = 0.72). This confirms that at least from the heart rate marker, the depth of anesthesia was consistent across ZT and pre-post conditions. One additional (17th) animal from the ZT13 group failed to maintain normal body temperature during the experiment and was excluded from the analysis. Each of the remaining recordings included simultaneous medial and lateral striatal (MStr and LStr) recordings and cerebellar Crus2/Paramedian lobule (CB) recordings. As explained above, in 4 of these animals, one electrode from each pair aimed at the MStr and LStr was instead placed in the overlying neocortex. Electrode locations determined from electrolytic lesion sites confirmed the location of microelectrode tips in the striatum and granule cell layer of the cerebellum or close vicinity. Placement of 17 of the 32 cerebellar pairs, 10 of the 16 MStr and LStr pairs and all of the neocortical electrode tips are shown in Figure 1, providing a representative spread of recording location for each brain area.

Both the striatal and cerebellar LFPs showed predominant large amplitude slow wave activity between 0.8–2 Hz with intermittent smaller amplitude oscillations around 4–5 Hz (Figure 2A). The slow wave activity is consistent with other findings in which urethane anesthesia induces widespread cortical synchrony resulting in oscillations at ~1 Hz that are intermittently interrupted by a desynchronized state (Destexhe et al., 1999; Clement et al., 2008). Slow wave activity tended to co-occur in the striatum and cerebellum, consistent with findings that anesthesia induces coherent slow-wave oscillations between the neocortex and striatum (Tseng et al., 2001; Mallet et al., 2005) and between the neocortex and cerebellum (Ros et al., 2009). We also observed transient spindle-like 8–20 Hz oscillations that were nested within the slow-wave activity. These spindles were most evident in the striatal LFPs, but similar electrophysiological events also occurred in the cerebellar LFPs (Figure 2B).

### Diurnal modulation of LFP oscillations

To determine the effects of time of day on both slow-wave and 3–8 Hz oscillations, the power of the FFT within each band recorded at baseline, was compared across the four times of day (Figure 3A). The MStr and LStr recordings showed the same variations by time of day and were thus combined for statistical analysis. In both striatal and cerebellar LFPs, slow-wave power showed a large diurnal rhythm in which power was the greatest at ZT1 and the lowest at ZT13 (Str, *F*_(3,52)_ = 11.80, *p* < 0.001; CB, *F*_(3,60)_ = 14.75, *p* < 0.001). There was an inverse diurnal pattern in the power of 3–8 Hz oscillations, in which power was highest at ZT13 (Str, *F*_(3,52)_ = 5.78, *p* < 0.01; CB, *F*_(3,60)_ = 11.56, *p* < 0.001). No diurnal effect was found in frequency bands above 8 Hz for either region (not shown).

Because the overall FFT power at 0–3 Hz and 3–8 Hz were inversely related to each other by time of day, we also sought to determine the relationship between the two frequency bands within each 2-s window, and to quantify differences in time spent in slow oscillations vs. 3–8 Hz oscillations. The relationship between the power of slow waves vs. 3–8 Hz oscillations was assessed by plotting the percent power of the FFT within the 0–3 Hz band vs. the 3–8 Hz band for each of the 180 2-s windows sampled in baseline recordings (Figure 3B). There was a strong negative correlation between power in the two bands for all recording sessions (Figure 3B shows two examples of this negative correlation). The inverse relationship of the power at 0–3 Hz vs. 3–8 Hz within each 2-s window indicated that the two oscillatory modes did not co-occur. This was then used to create the threshold to detect the duration of 0–3 Hz or 3–8 Hz activity within each recording session. Since no diurnal effect was observed in bands above 8 Hz, these bands were excluded from this detection analysis.

### Effects of raclopride on diurnal variations in the occurrence of LFP oscillations

Diurnal rhythms in the power of the LFP were highly associated to the proportion of time spent within each oscillatory mode, and raclopride influenced this distribution in a time-of-day-dependent manner (Figure 4). The percentage of time spent preferentially in slow wave oscillations vs. 3–8 Hz oscillations was calculated for each recording period, and both cerebellar and striatal LFPs showed robust, inverse diurnal rhythms for the proportion of time spent in each band (see Figures 4C,D, closed symbols; 0–3 Hz: Str, *F*_(3,52)_ = 6.63, *p* < 0.001, CB, *F*_(3,60)_ = 11.85, *p* < 0.001; 3–8 Hz: Str, *F*_(3,52)_ = 7.78, *p* < 0.001, CB, *F*_(3,60)_ = 13.86, *p* < 0.001). At ZT1, a large proportion of time was spent in slow wave oscillations (86.9 ± 5.9% of the time for cerebellum and 94.2 ± 5.7% for striatum) and at ZT13 there was a much greater prevalence of 3–8 Hz oscillations in both the striatum and cerebellar cortex (57.7 ± 5.2% of the time for cerebellum and 40.0 ± 4.3% for striatum). Overall, both cerebellar and striatal LFPs showed similar diurnal effects in the proportion of time spent in each of the two frequency bands.

Since circadian PER2 rhythms in the striatum are dependent upon dopamine and D2 receptor activation (Hood et al., 2010), we assessed the effects of raclopride on LFP oscillations as a function of time of day. For both striatal and cerebellar LFPs, systemic raclopride administration resulted in an increase in slow oscillations a decrease in in 3–8 Hz oscillations (Figures 4A,B). This effect was only significant at ZT13, except the 3–8 Hz band in the striatum, where only a trend was observed (Figures 4C,D; 0–3 Hz: Str, *F*_(3,52)_ = 3.00, *p* < 0.05; CB, *F*_(3,60)_ = 4.67, *p* < 0.01; 3–8 Hz: Str, *F*_(3,52)_ = 2.37, *p* = 0.08, CB, *F*_(3,60)_ = 11.91, *p* < 0.001). The raclopride-induced increase in the occurrence of 0–3 Hz oscillations at ZT13 was also mirrored in the overall power of the FFT (not shown). Typically, the changes in oscillatory activity were complementary between the bands, such that raclopride induced an approximate 15% increase in time spent in the 0–3 Hz band and an approximate 15% decrease in time spent in the 3–8 Hz band for both the striatal and cerebellar LFPs.

### Diurnal variation in the incidence of spindles

Spindles occur during slow wave oscillations during sleep and under urethane anesthesia, and can be modulated by distinct thalamic mechanisms (Valencia et al., 2013). We sought to determine if the incidence of spindles varies in a diurnal manner. The incidence of spindles was determined for striatal and cerebellar recording sites for each animal, and then compared between ZT and before and after raclopride administration. The occurrence of spindles in the LFP showed a similar pattern to that of slow waves, with a significant diurnal effect for striatal (*F*_(3,28)_ = 4.93, *p* < 0.01), but not cerebellar recordings (*F*_(3,12)_ = 2.12, *p* = 0.15) (Figure 5B). The diurnal pattern of spindles was dependent upon the occurrence of slow waves, such that when normalized to the amount of time spent in 0–3 Hz oscillations there was no diurnal variation in spindle incidence (Str, *F*_(3,28)_ = 1.16, *p* = 0.34; CB, *F*_(3,12)_ = 1.54, *p* = 0.25) (Figure 5C). In addition, raclopride did not have an effect on the incidence of spindles (Str, *F*_(3,28)_ = 1.17, *p* = 0.34; CB, *F*_(3,12)_ = 1.88, *p* = 0.20; data not shown). Therefore, diurnal variations in the incidence of spindles are dependent on the presence of slow wave oscillations under our urethane anesthesia preparation, and are not significantly affected by D2 receptor antagonism.

### Effects of raclopride on diurnal variations in coherence

**Figure 6:**
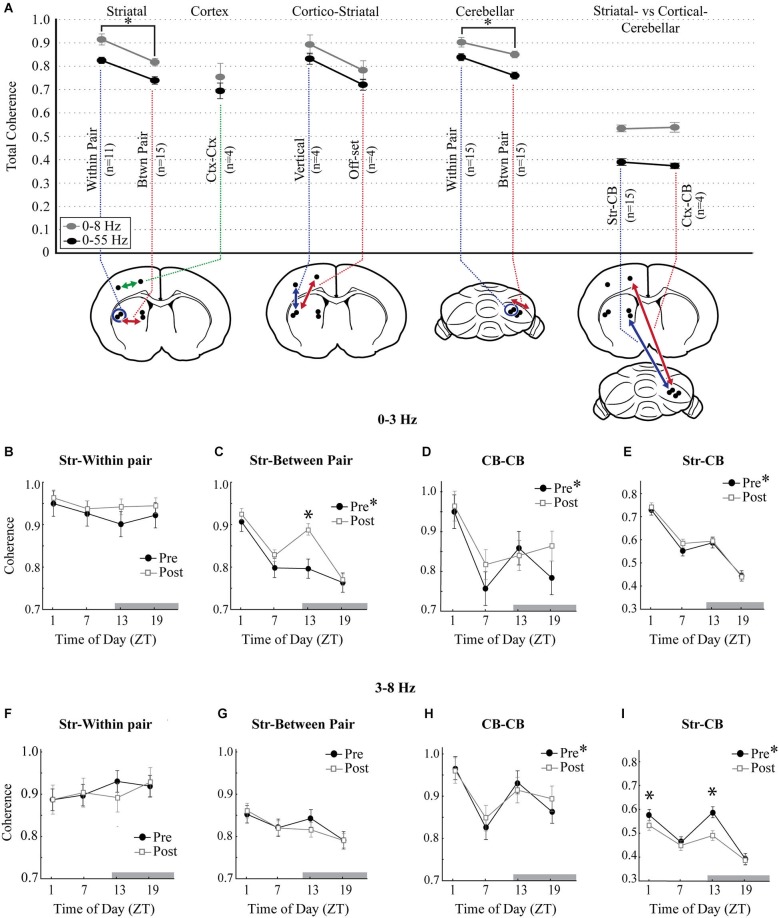
**Diurnal variation in measures of coherence**. **(A)** Overall total coherence of oscillations (0–8 Hz, gray circles; 0–55 Hz, black circles), averaged across all times of day and all animals. Coherence comparisons are indicated by arrows in the schematic diagrams (*n*, number of animals). “Within Pair” indicates comparisons within the electrode pairs, “Btwn Pair” indicates comparisons between more distant electrodes. Coherence comparisons indicated with different colors. Asterisks signify significance in the Tukey *post hoc* analysis, *p* < 0.05. **(B–E)** 0–3 Hz coherence measured across each of the striatal and cerebellar comparisons showing diurnal variations at baseline (filled circles) and the effects of raclopride (open squares). **(F–I)** Coherence measures at baseline and after raclopride in the 3–8 Hz band, across each of the striatal and cerebellar comparisons. Asterisks in the figure legend indicates a main effect of ZT at baseline (pre-raclopride), asterisks above the data points indicate significant effects of raclopride in the Tukey *post hoc* analysis, *p* < 0.05.

To assess the overall interregional differences in the synchrony of LFP oscillations recorded in the striatum and cerebellum, coherence within and between these structures was evaluated. Within the 0–3 Hz and 3–8 Hz bands, coherence showed similar levels and were therefore combined. There was also no difference between the following comparisons, so this data was combined: MStr and LStr within pair, the MStr-CB and LStr-CB, the two cortical locations with the CB, and the two off-set cortico-striatal comparisons. 0–8 Hz coherence followed a similar pattern as 8–55 Hz coherence, but was consistently higher (see Figure 6A, 0–8 Hz vs. 0–55 Hz). Coherence was higher for recordings obtained from adjacent tips within each of the striatal electrode pairs (Within Pair) as compared to recordings obtained between electrode pairs (Btwn Pair) in the MStr vs. LStr (*p* < 0.01) or across electrode pairs in the cerebellum (*p* < 0.001). This demonstrates enhanced coherence of LFPs with spatial proximity in both the striatum and the cerebellar cortex (Figure 6A, Within Pair vs. Btwn Pair comparisons). Four additional recording sessions were performed to evaluate the contribution of the neocortex to the striato-cerebellar coherence and included simultaneous recordings from two striatal electrodes, two electrodes in the overlying neocortex, and four electrodes in the cerebellar cortex. Cortico-striatal coherence was greatest between electrodes in the same sagittal plane and was comparable to the striatal-within pair comparisons (Figure 6A, 0–8 Hz: Str-within pair = 0.92 ± 0.024; Ctx-Str vertical = 0.90 ± 0.042). Cortico-striatal coherence was reduced for comparisons in different medial vs. lateral positions, showing a similar coherence as the striatal-between pair comparisons (Str-between pair = 0.82 ± 0.016; Ctx-Str off-set = 0.79 ± 0.042). The levels of Str-CB or Ctx-CB coherence (Str-CB = 0.53 ± 0.008; Ctx-CB = 0.54 ± 0.021) were substantially lower than levels of coherence obtained from more nearby electrodes pairs within the striatum or cerebellum. However, levels of both Str-CB and Ctx-CB coherence had similar values. This is consistent with the neocortex playing a role in coordinating oscillations in the striatum and cerebellum under urethane anesthesia.

The presence of diurnal effects on mean coherence for each recording session was assessed for the 0–3 Hz and 3–8 Hz bands within striatal, cerebellar and striato-cerebellar comparisons. CB within pair and between pair along with CB-MStr and CB-LStr comparisons showed the same modulation by time-of-day and pre-post raclopride, so these data were combined. Coherence of striatal LFPs showed a significant main effect for time-of-day for slow oscillations between medial and lateral recording sites (Str-between pair *F*_(3,48)_ = 7.24, *p* < 0.001) but not with closer electrode pairs (Str-within pair, *F*_(3,20)_ = 0.38, *p* = 0.77) demonstrating higher levels at ZT1 and lower levels at ZT13 (Figures 6B,C, closed symbols). However, there was no diurnal effect on coherence of 3–8 Hz oscillations (Figures 6F,G; Str-Within pair, *F*_(3,20)_ = 0.59, *p* = 0.63, Str-between pair *F*_(3,48)_ = 1.75, *p* = 0.17). Similar to the coherence of striatal slow waves, both cerebellar coherence and striato-cerebellar coherence within 0-3 Hz demonstrated a significant peak at ZT1 (Figures 6D,E; CB, *F*_(3,92)_ = 4.20, *p* < 0.01; Str-CB, *F*_(3,220)_ = 27.86, *p* < 0.001) which is consistent with the prevalence of slow oscillations in these structures at ZT1. Similarly, within the 3–8 Hz band, there was a strong diurnal pattern in cerebellar and striato-cerebellar coherence with increased coherence at ZT13 (Figures 6H,I; CB-CB, *F*_(3,92)_ = 5.54, *p* < 0.01; Str-CB, *F*_(3,220)_ = 16.54, *p* < 0.001) when both structures show predominant 3–8 Hz oscillations (Figure 4C). These comparisons also showed a secondary increase at ZT1, suggesting a biphasic pattern of coherence in the 3–8 Hz band.

To assess the effect of D2 receptor antagonism on striatal and cerebellar coherence, the mean coherence before and after raclopride administration was compared for each of the four times of day. Raclopride had the effect of increasing coherence of slow oscillations at ZT13 between the MStr and LStr only (Str-between pair, *F*_(3,44)_ = 7.52, *p* < 0.001). Raclopride minimally affected 3–8 Hz coherence, where it induced a decrease only in the striato-cerebellar comparisons at ZT1 and ZT13 (*F*_(3,220)_ = 11.291, *p* < 0.001, Figures 6F–I). In general, changes in coherence in 0–3 Hz oscillations followed a diurnal pattern that is similar to the overall FFT power within these bands (compare Figures 4C and 6). This implies that D2 receptor antagonism has the effect of enhancing both the power and coherence of slow oscillations, in a manner that is more potent at ZT13.

## Discussion

This study was aimed at determining if the neural network activity of the basal ganglia and the cerebellum, and their coupling, are influenced by time-of-day, and how raclopride, a D2 dopamine receptor antagonist, affects those relationships. A marked diurnal change in the rhythmicity of the striatal and cerebellar cortex networks was observed, with slow oscillatory activity decreasing at the beginning of the dark phase (when the rat would usually be more active), and 3–8 Hz activity increasing in the same period. The diurnal patterns of oscillations in the slow vs. 3–8 Hz bands were generally inversed, particularly in the cerebellum where 3–8 Hz oscillations were more prominent. This diurnal variation was also seen in the incidence of spindle activity, which was strongly influenced by the presence of the slow wave component. In addition, the shifting of activity from slow to 3–8 Hz at ZT13 was decreased by the administration of raclopride, which promoted slow band activity. Lastly, LFP coherence showed that cerebellar, striatal between-pair and striato-cerebellar slow wave coherence showed a strong diurnal pattern of modulation. Raclopride administration also influenced striatal coherence in a time-of-day dependent manner that was consistent with the shifts in oscillatory activity occurring in this area, such that coherence was enhanced when slow wave activity increased. These results show a clear diurnal modulation of striatal and cerebellar network activities and their coupling, and a time-specific effect of dopamine transmission that may serve to sculpt the activity of these networks.

### Oscillatory activity and coupling under urethane anesthesia

Our experimental preparation, using urethane anesthesia, was chosen in order to minimize the chance of LFP signal contamination by behavioral artifacts such as neural movement-related LFP signal changes. Using the conventional parameters of heart rate and foot-pinch reflex, depth of anesthesia was stable across conditions. Urethane anesthesia is only minimally depressive, and thus permissive to neural oscillations (Maggi and Meli, 1986; Steriade, 2003; Clement et al., 2008). As such, urethane could be used for studying circadian effects on oscillations and communication between networks. This preparation favors slow wave oscillations and widespread synchrony (circa 1 Hz), while permitting transient oscillations in the 3–8 Hz range. In the context of sleep and urethane anesthesia, these slow oscillations have sculpting features in the neocortex (Steriade, 2003; Clement et al., 2008) whereby cortical “up” and “down” states greatly influence larger scale networks (Wilson and Kawaguchi, 1996; Stern et al., 1998; Ros et al., 2009). This cortical influence helps local circuits maintain synchronized activity during sleep, or sleep-like states (Sanchez-Vives and McCormick, 2000). We observed that cortical LFPs had a close relationship with striatal LFPs, and were more dissimilar with the cerebellar LFPs (Figure 6). Still, Ctx-CB coherence between 0–3 Hz could reach above 0.7, potentially indicating a strong cortical influence. In both structures, cortical slow oscillations can influence the cell membrane potential and firing pattern: slow oscillations are indeed related to granule and Golgi firing, and Purkinje cell complex spikes (Ros et al., 2009). Slow oscillations also have a strong influence on neuronal excitability in the striatum (Wilson and Kawaguchi, 1996; Sharott et al., 2012). The slow oscillations that we observed, synchronized in the striatum and cerebellar cortex, are therefore likely to arise from a common contribution of the neocortex, which may drive activity in both regions. Spindles recorded under urethane anesthesia are under the influence of thalamic pacemakers, particularly the reticular thalamic nucleus (Steriade, 2003). They occur during cortical “up” states and are dependent on the corticothalamic inputs. From our results, it appears that the diurnal modulation was more driven by the presence of the slow oscillations, further confirming the potential extrinsic influence on the local striatal and cerebellar cortex networks.

Oscillatory interactions between the striatum and cerebellum could, however, involve a variety of reverberating circuits. The faster 3–8 Hz oscillations appear less uniform in their expression across recording sites and may be generated by more local processes that could be differentially organized in the cerebellum and striatum (Schnitzler and Gross, 2005). In the cerebellar cortex, the granule and Golgi cells seem to follow 5–30 Hz oscillations in a steady manner (Courtemanche et al., 2002; Dugué et al., 2009). However, faster 10–25 Hz oscillations occur in the awake primate and are stopped by movement (Pellerin and Lamarre, 1997; Courtemanche et al., 2002); they can also be synchronized with the neocortex under specific behavioral conditions (Courtemanche and Lamarre, 2005). These oscillations follow a parasagittal modulatory pattern (Courtemanche et al., 2009), adapting to the demands of the movement being performed. As for the striatum, both 4–12 Hz oscillations in rodents, and 10–30 Hz oscillations in primates also adapt to the task demands (Courtemanche et al., 2003; DeCoteau et al., 2007a; Thorn et al., 2010). This provides evidence of a capacity of the faster oscillations, and their synchrony, to adapt to behavioral conditions, organizing the networks in a task-dependent manner with a potentially more local organization than the slow oscillations.

### Diurnal and dopamine modulation of LFP oscillations

Many biological processes and daily changes in behavior are influenced by circadian gene expression patterns, yet little is known about the neural mechanisms that may be driving these changes. The striatum and the cerebellum are important subcortical structures for a number of functions subserving behavior, including motivation, adaptability of motor control, sequencing, and elaboration of cognitive and mental programs (Ito, 2006; Graybiel, 2008). To the best of our knowledge, this study is the first time subcortical LFP network activity has been analyzed in a circadian context. Our findings are consistent with the diurnal modulation of EEG oscillations observed in other brain sites (Grasing and Szeto, 1992), even though in this latter study, both sleep and awake states were included in their analysis. When states of sleep vs. wakefulness are compared, an increase in slow wave activity and inter-regional synchrony is expected early in the light phase (ZT1) when the rat would be falling asleep and, conversely, the slow oscillations should give way to faster oscillations in the transition into the dark phase (ZT13), when the animals become more active (Buzsáki and Draguhn, 2004). We found here that, even under urethane anesthesia, in both the striatum and the cerebellum, slow wave oscillations showed a clear diurnal variation that was consistent with this sleep-wake cycle. This indicates that the circadian mechanisms are robust enough to control network oscillations under anesthesia, suggesting that circadian clocks may influence how signal transmission occurs between local networks and play a role in establishing neural conditions that allow behavioral states to change throughout the day. The acceleration of the LFP oscillations and the shifts in coherence at the beginning of the dark period, at a time when the animal would normally be transitioning into its active phase, could be establishing optimal neural conditions that create a permissive state for networks to perform their awake activities.

Changes in neurotransmitter availability influence neuronal excitability and can contribute to the switching between oscillatory modes (McCormick, 2004), where different neuromodulators can promote shifts in frequencies in a manner that is anatomically specific (Roopun et al., 2010). Dopamine is known to promote wakefulness, but has scarcely been studied in its effect on cortical excitability during slow wave activity and our results seem similar to the neuromodulatory effects of dopamine in the entorhinal cortex (Mayne et al., 2013; Zeitzer, 2013). We found here that blocking D2 receptors could increase the slow oscillatory content of the LFP signal. Raclopride injections enhanced slow striatal and cerebellar oscillations, particularly at ZT13. This is the time of day that has the greatest reduction on the slow oscillations. This result points to a role of dopamine as a neuromodulator of the slow oscillations.

Dopamine itself has a strong influence on striatal oscillatory properties (Costa et al., 2006). Under urethane anesthesia, dopamine depletion increases basal ganglia entrainment to cortical slow oscillations (Tseng et al., 2001; Walters et al., 2007). Lesion to substantia nigra dopamine neurons causes depolarization of striatal medium spiny neurons and increases their spontaneous burst firing at frequencies coherent with the neocortex (Tseng et al., 2001; Sharott et al., 2012). This results in a decrease in the striatal filtering of the slow components from the neocortex, consequently increasing slow wave activity throughout the basal ganglia. Therefore, in our experiments, the decrease in slow wave activity in the striatum at ZT13 could be due to an increase in dopamine transmission. This is supported by raclopride having the largest effects at this time. In contrast, peak extracellular dopamine concentrations occur in the striatum somewhat later in the dark phase, around ZT18 (Owasoyo et al., 1979; Hood et al., 2010; Ferris et al., 2014). This suggests that oscillatory activity is being affected by factors in addition to dopamine availability, such as membrane receptor activation. Diurnal variations in the striatal LFP are likely dependent, in part, on alteration of dopamine function, which may help to provide an oscillatory state in the striatum which is more “disconnected” from the neocortex during awake conditions, thus enhancing the occurrence of higher frequency oscillations.

### Network interactions

In the cerebellar LFPs, we found slow oscillations that are thought to have a neocortical origin (Ros et al., 2009), and 3–8 Hz oscillations, which are likely dependent on reverberating properties in the cerebellar granule cell layer (Dugué et al., 2009; Courtemanche et al., 2013). The cerebellum followed a similar diurnal pattern in oscillations as the striatum (mostly slow oscillations at ZT1; fewer slow oscillations and more 3–8 Hz at ZT1) and its oscillatory profile was similarly affected by raclopride. While the oscillations recorded could in part come from local cerebellar circuit resonance (Dugué et al., 2009), it is quite possible that there are contributions from larger circuit interactions, including the subcortical connections between the basal ganglia and the cerebellum and the neocortico-ponto-cerebellar circuits.

Addressing the local circuits, it is unclear exactly how dopamine can affect cerebellar cortex circuitry. Even if not traditionally considered as a prominent cerebellar neuromodulator, there is evidence for dopaminergic transmission in the cerebellum (Takada et al., 1993; Hurley et al., 2003; Delis et al., 2004; Schweighofer et al., 2004; Giompres and Delis, 2005) There is also some evidence that dopamine levels in the cerebellum follow a circadian cycle (Owasoyo et al., 1979). However, the role of dopamine in the cerebellum remains poorly understood (Schweighofer et al., 2004). D2 and D3 type dopamine receptors have primarily been reported in the molecular cell layer of the vermis (Bouthenet et al., 1987), however raclopride does show low levels of binding to the cerebellar hemispheres *in vivo* (Kiss et al., 2011). Dopamine projections from the ventral tegmental area (VTA) to the cerebellar cortex have been reported to terminate in the granule and Purkinje cell layers in the posterior lobe (Ikai et al., 1992) and alterations of activity in the VTA both acutely and chronically induce changes in *cFOS* expression in the granule cell layer in multiple cerebellar regions, including Crus1 and 2 (Herrera-Meza et al., 2014). It may be that these VTA projections were affected by the systemic raclopride injections, altering neural activity in the areas we recorded, however the effects of dopamine on neural activity in the cerebellar cortex is also not well described. It is unknown if dopamine affects the resonance aspect in the cerebellar circuits, enhancing the 3–8 Hz resonance that appears optimal in those circuits, reducing the capacity to entrain local circuits to the 0–3 Hz inputs arriving from the cortex via the pontine nuclei. We have seen interactions between the GCL and the Purkinje cell layers in monkey ~15 Hz resonance, so faster oscillations could be partially affected by this local connectivity (Courtemanche et al., 2013). A potential mechanism could be the Lugaro connection affecting the Golgi cells, but at this point, since sufficient studies have not addressed this, these effects remains speculative.

A more probable action of dopamine in the cerebellar cortex however is its indirect effects through cerebellar interactions with other brain areas. The cerebellum is likely to interact with the striatum through the neocortex and subcortical connections. Interconnections between the neocortex and striatum as well as those between the neocortex and cerebellum are strong and numerous. Cortico-striatal connections are important inputs to the striatum (Graybiel, 2010), and these interactions are affected in disease models known to afflict the basal ganglia (Crittenden and Graybiel, 2011). In addition, cortico-pontocerebellar connections have an important effect on cerebellar processing (Morissette and Bower, 1996). This pathway constitutes one of the fastest central pathway in the CNS (Allen and Tsukahara, 1974). Overall, the potential avenue of communication between the striatum and cerebellar cortex taking advantage of the neocortical loops is a definite possibility. In addition, this interconnection is supplemented by the more recent finding of a plurisynaptic subcortical route between the cerebellum and basal ganglia (Hoshi et al., 2005; Bostan et al., 2010). In these papers and in their review, Bostan and Strick (2010) identify that these pathways permit bidirectional information transfer from the basal ganglia to the cerebellum (via a connection from the subthalamic nucleus to the pontine nuclei), or vice-versa (via a connection from the dentate nucleus to the thalamus—specifically, the thalamic central lateral nucleus (Ichinohe et al., 2000)), without requiring the passage through cerebral cortex connections. This anatomical connectivity is supported both by molecular and behavioral studies reporting alterations in either the basal ganglia or the cerebellum inducing rapid changes in the other area (Koch et al., 2009; Calderon et al., 2011; Moers-Hornikx et al., 2011). Since functional changes appear to be bidirectional, one would also assume changes in neural activity to have a similar bidirectional influence. Electrical stimulation of the dentate nucleus can increase firing in the basal ganglia (Li and Parker, 1969; Ratcheson and Li, 1969), suggesting that altered firing in the cerebellar cortex could also be produced by modifying the neural activity in the striatum.

Dopamine may also affect circadian changes in oscillatory activity by altering more widespread circuit properties, such as thalamocortical circuits, which could then cascade down an effect on both the cerebellar and striatal oscillations. This is consistent with findings that systemic D2 antagonism reduces wakefulness and increases slow wave sleep, where it is believed to act through the nuclei responsible for regulating sleep and wakefulness in the brainstem, hypothalamus, and basal forebrain (Monti and Monti, 2007). With our particular LFP patterns showing widespread synchrony ~1 Hz, and this oscillation having a strong link with the neocortex in the cerebellum (Ros et al., 2009) and striatum (Stern et al., 1998), it seems most likely that the coordination of the signals we recorded implicates neocortical activity. This would also support the appearance of spindles in the cerebellar LFPs. Spindles originate in the reticular thalamic and neocortical loops under conditions of slow wave oscillations, once appearing in the neocortex they could be transmit to the cerebellar cortex via the cortico-pontocerebellar pathway. It is possible that the neocortex circuits are also involved in the transmission of the 4–12 Hz activity between sites; many cortical areas do exhibit this 4–12 Hz activity, including the hippocampus (Buzsáki, 2006; Buzsáki and Moser, 2013), the cerebral somatosensory cortex (Nicolelis et al., 1995; Ahissar et al., 1997), and also the prefrontal cortex (Watson et al., 2014). However, this activity is better controlled locally, and less driven by large cortico-thalamic interactions that synchronize highly converging neocortical structures, as seems to be the case for slow rhythms during urethane anesthesia or sleep (Steriade, 2003).

The diurnal alterations between slow and 3–8 Hz oscillations are therefore likely influenced by the balance between widespread synchrony from the thalamocortical networks driving the slow oscillations and mechanisms underlying more locally generated oscillations. This is partly evidenced by the fact that when placing the electrodes, the cerebellum consistently showed weaker slow oscillations and stronger 3–8 Hz oscillations as compared to the striatum, reflective of a potentially more distant connection to the neocortex and stronger local resonance of this 3–8 Hz frequency range. This regional aspect would provide some rationale for the favoring of subcortical connections in the synchronization of theta-like activity patterns. A limitation of this study however, is that cortical activity can only be inferred in its role for driving these oscillations in both areas and to decipher regional connectivity will necessitate a more direct approach on the connectivity.

### Functional and clinical implications

This study uncovered a diurnal modulation that emphasizes the capacity of the striatal and cerebellar circuits to reorganize during the course of the day. The electrophysiological evidence presented here, with oscillations and synchrony alternating between ~1 Hz or ~4 Hz rhythms, shows that neural properties are being modulated in order to switch the underlying network states. This could allow the networks to optimally engage in various behaviors throughout the day. The drive in the diurnal modulation of these signals likely comes from the SCN, the central circadian pacemaker, and the subsidiary clocks that exist in a hierarchical network throughout the brain, including the striatum and the cerebellum (Namihira et al., 1999; Shieh, 2003; Imbesi et al., 2009; Rath et al., 2012; Harbour et al., 2013). Both latter areas are involved in locomotor activities driven by circadian gene expression (Masubuchi et al., 2000; Hood et al., 2010; Mendoza et al., 2010) and the diurnal modulation of LFP activity described here is likely to reflect network processes that promote these locomotor/movement activities and may further be important for fine-tuning sensorimotor performance and learning throughout the day. Future directions will include making the link between daily changes in neural network activity and behavioral processes that are driven by circadian genes.

There are a few neurological clinical conditions that affect both the basal ganglia and the cerebellum, and which display diurnal variations in symptoms or in response to pharmacological treatments. For example, Parkinson’s disease patients with motor symptom fluctuations generally experience the least amount of impairment in the morning, with symptoms increasing throughout the day (Nutt et al., 1997; Bruguerolle and Simon, 2002). In addition, Parkinson’s patients receiving dopamine treatments such as levodopa also experience a diurnal worsening of symptoms. Similarly, spinocerebellar type 3 ataxic symptoms also appear to show diurnal variations in symptoms (Wilder-Smith et al., 2003), though this seems to interact with a dyskinesia that is also levodopa responsive. The link could be direct with core function in these networks, as the ATX2 (ATAXIN-2) protein, which is implicated in the expression of spinocerebellar ataxia type 2 (or an increased risk of amyotrophic lateral sclerosis and Parkinsonism), is involved in the activation of the rate-limiting circadian clock component PERIOD in *Drosophila* (Lim and Allada, 2013) and their daily locomotor behavior (Zhang et al., 2013). In terms of circuit mechanisms, Nutt et al. (1997) report that circadian fluctuations in motor symptoms are independent of plasma levels of levodopa. Even when administering constant-rate infusions, symptoms still worsen despite plasma levels increasing throughout the day. This suggests that dopamine availability is not the only factor that is determining variations in motor behavior, but other factors such as receptor activation and interactions with other neuromodulators also likely play a role. Our results suggest that membrane targets responsible for changes in circuit properties change throughout the day, rather than simply availability of dopamine at the synapse. These daily alterations in circuit properties, both dopamine-related and otherwise, could underlie the diurnal symptomatology in basal ganglia and cerebellar patients.

## Author contributions

Ariana Frederick, Jonathan Bourget-Murray, C. Andrew Chapman, Shimon Amir and Richard Courtemanche conceived the project design. Shimon Amir and Richard Courtemanche supervised the project. Ariana Frederick and Richard Courtemanche collected all electrophysiology data. Ariana Frederick, Jonathan Bourget-Murray, and Richard Courtemanche analyzed and interpreted data. Ariana Frederick, Jonathan Bourget-Murray, C. Andrew Chapman, Shimon Amir and Richard Courtemanche contributed to the preparation of the manuscript.

## Conflict of interest statement

The authors declare no external commercial or financial relationships that could be perceived as a conflict of interest.

## References

[B1] Ahissar E. Haidarliu S. Zacksenhouse M. (1997). Decoding temporally encoded sensory input by cortical oscillations and thalamic phase comparators. Proc. Natl. Acad. Sci. U S A 94, 11633–11638 10.1073/pnas.94.21.11633 9326662 PMC23560

[B2] Albrecht U. Bordon A. Schmutz I. Ripperger J. (2007). The multiple facets of Per2. Cold Spring Harb. Symp. Quant. Biol. 72, 95–104 10.1101/sqb.2007.72.001 18419266

[B3] Allen G. I. Tsukahara N. (1974). Cerebrocerebellar communication systems. Physiol. Rev. 54, 957–1006 4370744 10.1152/physrev.1974.54.4.957

[B4] Ballion B. Frenois F. Zold C. L. Chetrit J. Murer M. G. Gonon F. (2009). D2 receptor stimulation, but not D1, restores striatal equilibrium in a rat model of Parkinsonism. Neurobiol. Dis. 35, 376–384 10.1016/j.nbd.2009.05.019 19501163

[B5] Başar E. Başar-Eroglu C. Karakaş S. Schürmann M. (2001). Gamma, alpha, delta and theta oscillations govern cognitive processes. Int. J. Psychophysiol. 39, 241–248 10.1016/s0167-8760(00)00145-8 11163901

[B6] Berke J. D. Okatan M. Skurski J. Eichenbaum H. B. (2004). Oscillatory entrainment of striatal neurons in freely moving rats. Neuron 43, 883–896 10.1016/j.neuron.2004.08.035 15363398

[B7] Bostan A. C. Dum R. P. Strick P. L. (2010). The basal ganglia communicate with the cerebellum. Proc. Natl. Acad. Sci. U S A 107, 8452–8456 10.1073/pnas.1000496107 20404184 PMC2889518

[B8] Bostan A. C. Strick P. L. (2010). The cerebellum and basal ganglia are interconnected. Neuropsychol. Rev. 20, 261–270 10.1007/s11065-010-9143-9 20811947 PMC3325093

[B9] Bouthenet M. L. Martres M. P. Sales N. Schwartz J. C. (1987). A detailed mapping of dopamine D-2 receptors in rat central nervous system by autoradiography with [125I]iodosulpride. Neuroscience 20, 117–155 10.1016/0306-4522(87)90008-x 2882443

[B10] Brown P. Oliviero A. Mazzone P. Insola A. Tonali P. Di Lazzaro V. (2001). Dopamine dependency of oscillations between subthalamic nucleus and pallidum in Parkinson’s disease. J. Neurosci. 21, 1033–1038 11157088 10.1523/JNEUROSCI.21-03-01033.2001 PMC6762327

[B11] Bruguerolle B. Simon N. (2002). Biologic rhythms and Parkinson’s disease: a chronopharmacologic approach to considering fluctuations in function. Clin. Neuropharmacol. 25, 194–201 10.1097/00002826-200207000-00002 12151906

[B12] Buehlmann A. Deco G. (2010). Optimal information transfer in the cortex through synchronization. PLoS Comput. Biol. 6:e1000934 10.1371/journal.pcbi.1000934 20862355 PMC2940722

[B13] Buzsáki G. (2006). Rhythms of the Brain. New York: Oxford University Press

[B14] Buzsáki G. Draguhn A. (2004). Neuronal oscillations in cortical networks. Science 304, 1926–1929 10.1126/science.1099745 15218136

[B15] Buzsáki G. Moser E. I. (2013). Memory, navigation and theta rhythm in the hippocampal-entorhinal system. Nat. Neurosci. 16, 130–138 10.1038/nn.3304 23354386 PMC4079500

[B16] Calderon D. P. Fremont R. Kraenzlin F. Khodakhah K. (2011). The neural substrates of rapid-onset Dystonia-Parkinsonism. Nat. Neurosci. 14, 357–365 10.1038/nn.2753 21297628 PMC3430603

[B17] Chaudhury D. Wang L. M. Colwell C. S. (2005). Circadian regulation of hippocampal long-term potentiation. J. Biol. Rhythms 20, 225–236 10.1177/0748730405276352 15851529 PMC2581477

[B18] Clement E. A. Richard A. Thwaites M. Ailon J. Peters S. Dickson C. T. (2008). Cyclic and sleep-like spontaneous alternations of brain state under urethane anaesthesia. PLoS One 3:e2004 10.1371/journal.pone.0002004 18414674 PMC2289875

[B19] Colwell C. S. (2011). Linking neural activity and molecular oscillations in the SCN. Nat. Rev. Neurosci. 12, 553–569 10.1038/nrn3086 21886186 PMC4356239

[B20] Costa R. M. Lin S. C. Sotnikova T. D. Cyr M. Gainetdinov R. R. Caron M. G. (2006). Rapid alterations in corticostriatal ensemble coordination during acute dopamine-dependent motor dysfunction. Neuron 52, 359–369 10.1016/j.neuron.2006.07.030 17046697

[B21] Courtemanche R. Chabaud P. Lamarre Y. (2009). Synchronization in primate cerebellar granule cell layer local field potentials: basic anisotropy and dynamic changes during active expectancy. Front. Cell. Neurosci. 3:6 10.3389/neuro.03.006.2009 19649170 PMC2718782

[B22] Courtemanche R. Fujii N. Graybiel A. M. (2003). Synchronous, focally modulated beta-band oscillations characterize local field potential activity in the striatum of awake behaving monkeys. J. Neurosci. 23, 11741–11752 10.3410/f.1002999.201447 14684876 PMC6740936

[B23] Courtemanche R. Lamarre Y. (2005). Local field potential oscillations in primate cerebellar cortex: synchronization with cerebral cortex during active and passive expectancy. J. Neurophysiol. 93, 2039–2052 10.1152/jn.00080.2004 15590736

[B24] Courtemanche R. Pellerin J. P. Lamarre Y. (2002). Local field potential oscillations in primate cerebellar cortex: modulation during active and passive expectancy. J. Neurophysiol. 88, 771–782 10.1152/jn.00718.2001 12163529

[B25] Courtemanche R. Robinson J. C. Aponte D. I. (2013). Linking oscillations in cerebellar circuits. Front. Neural Circuits 7:125 10.3389/fncir.2013.00125 23908606 PMC3725427

[B26] Crittenden J. R. Graybiel A. M. (2011). Basal ganglia disorders associated with imbalances in the striatal striosome and matrix compartments. Front. Neuroanat. 5:59 10.3389/fnana.2011.00059 21941467 PMC3171104

[B27] Darna M. Schmutz I. Richter K. Yelamanchili S. V. Pendyala G. Holtje M. (2009). Time of day-dependent sorting of the vesicular glutamate transporter to the plasma membrane. J. Biol. Chem. 284, 4300–4307 10.1074/jbc.m805480200 19103593

[B28] DeCoteau W. E. Thorn C. Gibson D. J. Courtemanche R. Mitra P. Kubota Y. (2007a). Learning-related coordination of striatal and hippocampal theta rhythms during acquisition of a procedural maze task. Proc. Natl. Acad. Sci. U S A 104, 5644–5649 10.1073/pnas.0700818104 17372196 PMC1838454

[B29] DeCoteau W. E. Thorn C. Gibson D. J. Courtemanche R. Mitra P. Kubota Y. (2007b). Oscillations of local field potentials in the rat dorsal striatum during spontaneous and instructed behaviors. J. Neurophysiol. 97, 3800–3805 10.1152/jn.00108.2007 17329629

[B30] Dejean C. Arbuthnott G. Wickens J. R. Le Moine C. Boraud T. Hyland B. I. (2011). Power fluctuations in beta and gamma frequencies in rat globus pallidus: association with specific phases of slow oscillations and differential modulation by dopamine D1 and D2 receptors. J. Neurosci. 31, 6098–6107 10.1523/JNEUROSCI.3311-09.2011 21508235 PMC6632973

[B31] Delis F. Mitsacos A. Giompres P. (2004). Dopamine receptor and transporter levels are altered in the brain of Purkinje cell degeneration mutant mice. Neuroscience 125, 255–268 10.1016/j.neuroscience.2004.01.020 15051164

[B32] Destexhe A. Contreras D. Steriade M. (1999). Spatiotemporal analysis of local field potentials and unit discharges in cat cerebral cortex during natural wake and sleep states. J. Neurosci. 19, 4595–4608 10341257 10.1523/JNEUROSCI.19-11-04595.1999 PMC6782626

[B33] Doya K. (2000). Complementary roles of basal ganglia and cerebellum in learning and motor control. Curr. Opin. Neurobiol. 10, 732–739 10.1016/s0959-4388(00)00153-7 11240282

[B34] Dugué G. P. Brunel N. Hakim V. Schwartz E. J. Chat M. Lévesque M. (2009). Electrical coupling mediates tunable low-frequency oscillations and resonance in the cerebellar Golgi cell network. Neuron 61, 126–139 10.1016/j.neuron.2008.11.028 19146818

[B35] Ferris M. J. Espana R. A. Locke J. L. Konstantopoulos J. K. Rose J. H. Chen R. (2014). Dopamine transporters govern diurnal variation in extracellular dopamine tone. Proc. Natl. Acad. Sci. U S A 111, E2751–E2759 10.1073/pnas.1407935111 24979798 PMC4084435

[B36] Fowler S. C. Liou J. R. (1998). Haloperidol, raclopride and eticlopride induce microcatalepsy during operant performance in rats, but clozapine and SCH 23390 do not. Psychopharmacology (Berl) 140, 81–90 10.1007/s002130050742 9862406

[B37] Frederick A. Bourget-Murray J. Courtemanche R. (2013). “Local field potential, synchrony of,” in Encyclopedia of Computational Neuroscience: Springer Reference, eds Jaeger D. Jung R. (Berlin Heidelberg: Springer-Verlag). Available online at: http://www.springerreference.com

[B38] Giompres P. Delis F. (2005). Dopamine transporters in the cerebellum of mutant mice. Cerebellum 4, 105–111 10.1080/14734220510007851 16035192

[B39] Grasing K. Szeto H. (1992). Diurnal variation in continuous measures of the rat EEG power spectra. Physiol. Behav. 51, 249–254 10.1016/0031-9384(92)90138-r 1557436

[B40] Gravotta L. Gavrila A. M. Hood S. Amir S. (2011). Global depletion of dopamine using intracerebroventricular 6-hydroxydopamine injection disrupts normal circadian wheel-running patterns and PERIOD2 expression in the rat forebrain. J. Mol. Neurosci. 45, 162–171 10.1007/s12031-011-9520-8 21484443

[B41] Graybiel A. M. (2008). Habits, rituals and the evaluative brain. Annu. Rev. Neurosci. 31, 359–387 10.1146/annurev.neuro.29.051605.112851 18558860

[B42] Graybiel A. M. (2010). “Templates for neural dynamics in the striatum: striosomes and matrisomes,” in Handbook of Brain Microcircuits, eds Shepherd G. M. Grillner S. (New York, NY: Oxford University Press), 120–126

[B43] Guilding C. Piggins H. D. (2007). Challenging the omnipotence of the suprachiasmatic timekeeper: are circadian oscillators present throughout the mammalian brain? Eur. J. Neurosci. 25, 3195–3216 10.1111/j.1460-9568.2007.05581.x 17552989

[B44] Harbour V. L. Weigl Y. Robinson B. Amir S. (2013). Comprehensive mapping of regional expression of the clock protein PERIOD2 in rat forebrain across the 24-h day. PLoS One 8:e76391 10.1371/journal.pone.0076391 24124556 PMC3790676

[B45] Herrera-Meza G. Aguirre-Manzo L. Coria-Avila G. A. Lopez-Meraz M. L. Toledo-Cárdenas R. Manzo J. (2014). Beyond the basal ganglia: cFOS expression in the cerebellum in response to acute and chronic dopaminergic alterations. Neuroscience 267, 219–231 10.1016/j.neuroscience.2014.02.046 24631673

[B46] Herzog E. D. (2007). Neurons and networks in daily rhythms. Nat. Rev. Neurosci. 8, 790–802 10.1038/nrn2215 17882255

[B47] Hood S. Cassidy P. Cossette M. P. Weigl Y. Verwey M. Robinson B. (2010). Endogenous dopamine regulates the rhythm of expression of the clock protein PER2 in the rat dorsal striatum via daily activation of D2 dopamine receptors. J. Neurosci. 30, 14046–14058 10.1523/JNEUROSCI.2128-10.2010 20962226 PMC6634752

[B48] Hoshi E. Tremblay L. Feger J. Carras P. L. Strick P. L. (2005). The cerebellum communicates with the basal ganglia. Nat. Neurosci. 8, 1491–1493 10.1038/nn1544 16205719

[B49] Hurley M. J. Mash D. C. Jenner P. (2003). Markers for dopaminergic neurotransmission in the cerebellum in normal individuals and patients with Parkinson’s disease examined by RT-PCR. Eur. J. Neurosci. 18, 2668–2672 10.1046/j.1460-9568.2003.02963.x 14622169

[B50] Hutcheon B. Yarom Y. (2000). Resonance, oscillation and the intrinsic frequency preferences of neurons. Trends Neurosci. 23, 216–222 10.1016/s0166-2236(00)01547-2 10782127

[B51] Ichinohe N. Mori F. Shoumura K. (2000). A di-synaptic projection from the lateral cerebellar nucleus to the laterodorsal part of the striatum via the central lateral nucleus of the thalamus in the rat. Brain Res. 880, 191–197 10.1016/s0006-8993(00)02744-x 11033006

[B52] Ikai Y. Takada M. Shinonaga Y. Mizuno N. (1992). Dopaminergic and non-dopaminergic neurons in the ventral tegmental area of the rat project, respectively, to the cerebellar cortex and deep cerebellar nuclei. Neuroscience 51, 719–728 10.1016/0306-4522(92)90310-x 1362601

[B53] Imbesi M. Yildiz S. Dirim Arslan A. Sharma R. Manev H. Uz T. (2009). Dopamine receptor-mediated regulation of neuronal “clock” gene expression. Neuroscience 158, 537–544 10.1016/j.neuroscience.2008.10.044 19017537 PMC3664916

[B54] Ito M. (2006). Cerebellar circuitry as a neuronal machine. Prog. Neurobiol. 78, 272–303 10.1016/j.pneurobio.2006.02.006 16759785

[B55] Kiss B. Horti F. Bobok A. (2011). In vitro and in vivo comparison of [^3^H](+)-PHNO and [^3^H]raclopride binding to rat striatum and lobes 9 and 10 of the cerebellum: a method to distinguish dopamine D(3) from D(2) receptor sites. Synapse 65, 467–478 10.1002/syn.20867 20936685

[B56] Koch G. Brusa L. Carrillo F. Lo Gerfo E. Torriero S. Oliveri M. (2009). Cerebellar magnetic stimulation decreases levodopa-induced dyskinesias in Parkinson disease. Neurology 73, 113–119 10.1212/WNL.0b013e3181ad5387 19597133

[B57] Lemaire N. Hernandez L. F. Hu D. Kubota Y. Howe M. W. Graybiel A. M. (2012). Effects of dopamine depletion on LFP oscillations in striatum are task- and learning-dependent and selectively reversed by L-DOPA. Proc. Natl. Acad. Sci. U S A 109, 18126–18131 10.1073/pnas.1216403109 23074253 PMC3497773

[B58] Li C. L. Parker L. O. (1969). Effect of dentate stimulation on neuronal activity in the globus pallidus. Exp. Neurol. 24, 298–309 10.1016/0014-4886(69)90023-5 5784137

[B59] Lim C. Allada R. (2013). ATAXIN-2 activates PERIOD translation to sustain circadian rhythms in Drosophila. Science 340, 875–879 10.1126/science.1234785 23687047

[B60] Maggi C. A. Meli A. (1986). Suitability of urethane anesthesia for physiopharmacological investigations in various systems. Part 1: general considerations. Experientia 42, 109–114 10.1007/bf01952426 2868911

[B61] Magill P. J. Sharott A. Bolam J. P. Brown P. (2004). Brain state-dependency of coherent oscillatory activity in the cerebral cortex and basal ganglia of the rat. J. Neurophysiol. 92, 2122–2136 10.1152/jn.00333.2004 15175372

[B62] Mallet N. Le Moine C. Charpier S. Gonon F. (2005). Feedforward inhibition of projection neurons by fast-spiking GABA interneurons in the rat striatum in vivo. J. Neurosci. 25, 3857–3869 10.1523/jneurosci.5027-04.2005 15829638 PMC6724938

[B63] Masubuchi S. Honma S. Abe H. Ishizaki K. Namihira M. Ikeda M. (2000). Clock genes outside the suprachiasmatic nucleus involved in manifestation of locomotor activity rhythm in rats. Eur. J. Neurosci. 12, 4206–4214 10.1111/j.1460-9568.2000.01313.x 11122332

[B64] Mayne E. W. Craig M. T. McBain C. J. Paulsen O. (2013). Dopamine suppresses persistent network activity via D(1) -like dopamine receptors in rat medial entorhinal cortex. Eur. J. Neurosci. 37, 1242–1247 10.1111/ejn.12125 23336973 PMC3628042

[B65] McCormick D. A. (2004). “Membrane properties and neurotransmitter actions,” in The Synaptic Organization of the Brain, ed Shepherd G. M. (New York, NY: Oxford University Press), 39–77

[B66] Mendoza J. Pevet P. Felder-Schmittbuhl M. P. Bailly Y. Challet E. (2010). The cerebellum harbors a circadian oscillator involved in food anticipation. J. Neurosci. 30, 1894–1904 10.1523/JNEUROSCI.5855-09.2010 20130198 PMC6634001

[B67] Middleton F. A. Strick P. L. (2000). Basal ganglia and cerebellar loops: motor and cognitive circuits. Brain Res. Brain Res. Rev. 31, 236–250 10.1016/s0165-0173(99)00040-5 10719151

[B68] Moers-Hornikx V. M. Vles J. S. Tan S. K. Cox K. Hoogland G. Steinbusch W. M. (2011). Cerebellar nuclei are activated by high-frequency stimulation of the subthalamic nucleus. Neurosci. Lett. 496, 111–115 10.1016/j.neulet.2011.03.094 21511005

[B69] Monti J. M. Monti D. (2007). The involvement of dopamine in the modulation of sleep and waking. Sleep Med. Rev. 11, 113–133 10.1016/j.smrv.2006.08.003 17275369

[B70] Mordel J. Karnas D. Pevet P. Isope P. Challet E. Meissl H. (2013). The output signal of Purkinje cells of the cerebellum and circadian rhythmicity. PLoS One 8:e58457 10.1371/journal.pone.0058457 23505510 PMC3591352

[B71] Morissette J. Bower J. M. (1996). Contribution of somatosensory cortex to responses in the rat cerebellar granule cell layer following peripheral tactile stimulation. Exp. Brain Res. 109, 240–250 10.1007/bf00231784 8738373

[B72] Namihira M. Honma S. Abe H. Tanahashi Y. Ikeda M. Honma K. (1999). Daily variation and light responsiveness of mammalian clock gene, Clock and BMAL1, transcripts in the pineal body and different areas of brain in rats. Neurosci. Lett. 267, 69–72 10.1016/s0304-3940(99)00324-9 10400251

[B73] Nicolelis M. A. L. Baccala L. A. Lin R. C. S. Chapin J. K. (1995). Sensorimotor encoding by synchronous neural ensemble activity at multiple levels of the somatosensory system. Science 268, 1353–1358 10.1126/science.7761855 7761855

[B74] Nutt J. G. Carter J. H. Lea E. S. Woodward W. R. (1997). Motor fluctuations during continuous levodopa infusions in patients with Parkinson’s disease. Mov. Disord. 12, 285–292 10.1002/mds.870120304 9159720

[B75] Owasoyo J. O. Walker C. A. Whitworth U. G. (1979). Diurnal variation in the dopamine level of rat brain areas: effect of sodium phenobarbital. Life Sci. 25, 119–122 10.1016/0024-3205(79)90382-5 573832

[B76] Paxinos G. Watson C. (1998). The Rat Brain in Stereotaxic Coordinates. SanDiego, CA: Academic Press

[B77] Pellerin J. P. Lamarre Y. (1997). Local field potential oscillations in primate cerebellar cortex during voluntary movement. J. Neurophysiol. 78, 3502–3507 9405570 10.1152/jn.1997.78.6.3502

[B78] Ratcheson R. A. Li C. L. (1969). Effect of dentate stimulation on neuronal activity in the caudate nucleus. Exp. Neurol. 25, 268–281 10.1016/0014-4886(69)90050-8 5345013

[B79] Rath M. F. Rohde K. Moller M. (2012). Circadian oscillations of molecular clock components in the cerebellar cortex of the rat. Chronobiol. Int. 29, 1289–1299 10.3109/07420528.2012.728660 23131067

[B80] Rivlin-Etzion M. Marmor O. Heimer G. Raz A. Nini A. Bergman H. (2006). Basal ganglia oscillations and pathophysiology of movement disorders. Curr. Opin. Neurobiol. 16, 629–637 10.1016/j.conb.2006.10.002 17084615

[B81] Roopun A. K. Lebeau F. E. Rammell J. Cunningham M. O. Traub R. D. Whittington M. A. (2010). Cholinergic neuromodulation controls directed temporal communication in neocortex in vitro. Front. Neural Circuits 4:8 10.3389/fncir.2010.00008 20407636 PMC2856628

[B82] Ros H. Sachdev R. N. Yu Y. Sestan N. McCormick D. A. (2009). Neocortical networks entrain neuronal circuits in cerebellar cortex. J. Neurosci. 29, 10309–10320 10.1523/JNEUROSCI.2327-09.2009 19692605 PMC3137973

[B83] Sanchez-Vives M. V. McCormick D. A. (2000). Cellular and network mechanisms of rhythmic recurrent activity in neocortex. Nat. Neurosci. 3, 1027–1034 10.1038/79848 11017176

[B84] Schnitzler A. Gross J. (2005). Normal and pathological oscillatory communication in the brain. Nat. Rev. Neurosci. 6, 285–296 10.1038/nrn1650 15803160

[B85] Schweighofer N. Doya K. Kuroda S. (2004). Cerebellar aminergic neuromodulation: towards a functional understanding. Brain Res. Brain Res. Rev. 44, 103–116 10.1016/j.brainresrev.2003.10.004 15003388

[B86] Sharma A. V. Wolansky T. Dickson C. T. (2010). A comparison of sleeplike slow oscillations in the hippocampus under ketamine and urethane anesthesia. J. Neurophysiol. 104, 932–939 10.1152/jn.01065.2009 20538775

[B87] Sharott A. Doig N. M. Mallet N. Magill P. J. (2012). Relationships between the firing of identified striatal interneurons and spontaneous and driven cortical activities in vivo. J. Neurosci. 32, 13221–13236 10.1523/jneurosci.2440-12.2012 22993438 PMC4242971

[B88] Sharott A. Magill P. J. Harnack D. Kupsch A. Meissner W. Brown P. (2005). Dopamine depletion increases the power and coherence of beta-oscillations in the cerebral cortex and subthalamic nucleus of the awake rat. Eur. J. Neurosci. 21, 1413–1422 10.1111/j.1460-9568.2005.03973.x 15813951

[B89] Shieh K. R. (2003). Distribution of the rhythm-related genes rPERIOD1, rPERIOD2 and rCLOCK, in the rat brain. Neuroscience 118, 831–843 10.1016/s0306-4522(03)00004-6 12710990

[B90] Steriade M. (2003). Neuronal Substrates of Sleep and Epilepsy. Cambridge, UK: Cambridge University Press

[B91] Stern E. A. Jaeger D. Wilson C. J. (1998). Membrane potential synchrony of simultaneously recorded striatal spiny neurons in vivo. Nature 394, 475–478 10.1038/28848 9697769

[B92] Takada M. Sugimoto T. Hattori T. (1993). MPTP neurotoxicity to cerebellar Purkinje cells in mice. Neurosci. Lett. 150, 49–52 10.1016/0304-3940(93)90105-t 8469402

[B93] Thorn C. A. Atallah H. Howe M. Graybiel A. M. (2010). Differential dynamics of activity changes in dorsolateral and dorsomedial striatal loops during learning. Neuron 66, 781–795 10.1016/j.neuron.2010.04.036 20547134 PMC3108575

[B94] Thorn C. A. Graybiel A. M. (2014). Differential entrainment and learning-related dynamics of spike and local field potential activity in the sensorimotor and associative striatum. J. Neurosci. 34, 2845–2859 10.1523/jneurosci.1782-13.2014 24553926 PMC3931500

[B95] Tseng K. Y. Kasanetz F. Kargieman L. Riquelme L. A. Murer M. G. (2001). Cortical slow oscillatory activity is reflected in the membrane potential and spike trains of striatal neurons in rats with chronic nigrostriatal lesions. J. Neurosci. 21, 6430–6439 11487667 10.1523/JNEUROSCI.21-16-06430.2001 PMC6763136

[B96] Uhlhaas P. J. Roux F. Rodriguez E. Rotarska-Jagiela A. Singer W. (2010). Neural synchrony and the development of cortical networks. Trends Cogn. Sci. 14, 72–80 10.1016/j.tics.2009.12.002 20080054

[B97] Valencia M. Artieda J. Bolam J. P. Mena-Segovia J. (2013). Dynamic interaction of spindles and gamma activity during cortical slow oscillations and its modulation by subcortical afferents. PLoS One 8:e67540 10.1371/journal.pone.0067540 23844020 PMC3699652

[B98] Verwey M. Amir S. (2012). Variable restricted feeding disrupts the daily oscillations of Period2 expression in the limbic forebrain and dorsal striatum in rats. J. Mol. Neurosci. 46, 258–264 10.1007/s12031-011ds-9529-z 21547532

[B99] Walters J. R. Hu D. Itoga C. A. Parr-Brownlie L. C. Bergstrom D. A. (2007). Phase relationships support a role for coordinated activity in the indirect pathway in organizing slow oscillations in basal ganglia output after loss of dopamine. Neuroscience 144, 762–776 10.1016/j.neuroscience.2006.10.006 17112675 PMC3354994

[B100] Watson T. C. Becker N. Apps R. Jones M. W. (2014). Back to front: cerebellar connections and interactions with the prefrontal cortex. Front. Syst. Neurosci. 8:4 10.3389/fnsys.2014.00004 24550789 PMC3912388

[B101] Wilder-Smith E. Tan E. K. Law H. Y. Zhao Y. Ng I. Wong M. C. (2003). Spinocerebellar ataxia type 3 presenting as an L-DOPA responsive dystonia phenotype in a Chinese family. J. Neurol. Sci. 213, 25–28 10.1016/s0022-510x(03)00129-1 12873751

[B102] Wilson C. J. Kawaguchi Y. (1996). The origins of two-state spontaneous membrane potential fluctuations of neostriatal spiny neurons. J. Neurosci. 16, 2397–2410 8601819 10.1523/JNEUROSCI.16-07-02397.1996 PMC6578540

[B103] Zeitzer J. M. (2013). Control of sleep and wakefulness in health and disease. Prog. Mol. Biol. Transl. Sci. 119, 137–154 10.1016/B978-0-12-396971-2.00006-3 23899597

[B104] Zhang Y. Ling J. Yuan C. Dubruille R. Emery P. (2013). A role for Drosophila ATX2 in activation of PER translation and circadian behavior. Science 340, 879–882 10.1126/science.1234746 23687048 PMC4078874

